# Orthomyxo-, paramyxo- and flavivirus infections in wild waterfowl in Finland

**DOI:** 10.1186/1743-422X-5-35

**Published:** 2008-02-28

**Authors:** Erika Lindh, Anita Huovilainen, Osmo Rätti, Christine Ek-Kommonen, Tarja Sironen, Eili Huhtamo, Hannu Pöysä, Antti Vaheri, Olli Vapalahti

**Affiliations:** 1 Department of Virology, Haartman Institute, Faculty of Medicine, P.O. Box 21, FI-00014 University of Helsinki, Finland; 2 Finnish Food Safety Authority Evira, Department of Animal Diseases and Food Safety Research, Virology Unit, Mustialankatu 3, FI-00790 Helsinki, Finland; 3 Arctic Centre, University of Lapland, P.O. Box 122, FI-96101 Rovaniemi, Finland; 4 Division of Microbiology and Epidemiology, Department of Basic Veterinary Sciences, Faculty of Veterinary Medicine, P.O. Box 66, FI-00014 University of Helsinki, Finland; 5 Finnish Game and Fisheries Research Institute, Joensuu Game and Fisheries Research, Yliopistonkatu 6, FI-80100 Joensuu, Finland; 6 Department of Virology, HUSLAB, Hospital District of Helsinki and Uusimaa, P.O. Box 400, FI-00029 HUS, Helsinki, Finland

## Abstract

**Background:**

Screening wild birds for viral pathogens has become increasingly important. We tested a screening approach based on blood and cloacal and tracheal swabs collected by hunters to study the prevalence of influenza A, paramyxo-, flavi-, and alphaviruses in Finnish wild waterfowl, which has been previously unknown. We studied 310 blood samples and 115 mixed tracheal and cloacal swabs collected from hunted waterfowl in 2006. Samples were screened by RT-PCR and serologically by hemagglutination inhibition (HI) test or enzyme-linked immunosorbent assay (ELISA) for influenza A (FLUAV), type 1 avian paramyxo-(APMV-1), Sindbis (SINV), West Nile (WNV) and tick-borne encephalitis (TBEV) virus infections.

**Results:**

FLUAV RNA was found in 13 tracheal/cloacal swabs and seven strains were isolated. Five blood samples were antibody positive. Six APMV-1 RNA-positive samples were found from which four strains were isolated, while two blood samples were antibody positive. None of the birds were positive for flavivirus RNA but three birds had flavivirus antibodies by HI test. No antibodies to SINV were detected.

**Conclusion:**

We conclude that circulation of both influenza A virus and avian paramyxovirus-1 in Finnish wild waterfowl was documented. The FLUAV and APMV-1 prevalences in wild waterfowl were 11.3% and 5.2% respectively, by this study. The subtype H3N8 was the only detected FLUAV subtype while APMV-1 strains clustered into two distinct lineages. Notably, antibodies to a likely mosquito-borne flavivirus were detected in three samples. The screening approach based on hunted waterfowl seemed reliable for monitoring FLUAV and APMV by RT-PCR from cloacal or tracheal samples, but antibody testing in this format seemed to be of low sensitivity.

## Background

*Influenza A virus *(FLUAV) is a member of the family *Orthomyxoviridae*, naturally hosted by wild waterfowl. All subtypes, composed by different combinations of the 16 hemagglutinin (HA) types and 9 neuraminidase (NA) types, have been isolated from birds but lineages of certain viruses are occasionally established in non-avian hosts including humans [1,2]. Most strains found in wild waterfowl are of the low-pathogenic avian influenza (LPAI) phenotype. Highly pathogenic (HPAI) phenotypes of H5 and H7 subtypes have increasingly caused disease outbreaks in poultry and the H5N1 type initially isolated in China has spread throughout Asia and into Europe and Africa infecting both poultry and wild birds [3]. The emergence of HPAI and the ecology of FLUAV in wild waterfowl have been reviewed elsewhere [4].

Occurence of influenza A viruses in wild birds has been monitored since 2003 in the EU including Finland. Although high prevalences of FLUAV in wild waterfowl have been reported from other Northern European countries [5,6] the previous Finnish findings of FLUAV infected birds are limited to a few viruses of the H13N6 subtype isolated from herring gulls in 2005 (Jonsson et al., manuscript in preparation) and to the isolation of an untyped FLUAV from a mallard in 1979 [7].

Newcastle disease (ND) in poultry is caused by type 1 of the nine species (designated *avian paramyxovirus 1–9*) in the genus *Avulavirus*, a member of the family *Paramyxoviridae *[8]. Avian paramyxovirus-1 (APMV-1) infects a wide range of bird species of different orders causing disease of varying severity. The strains are classified according to the pathogenicity in chickens and the deduced amino acid sequence of the cleavage site of the fusion protein into lentogenic (mildly virulent), mesogenic (intermediate virulence) and velogenic (highly virulent) strains [9]. Similar to FLUAV, velogenic strains of APMV-1 are suspected to arise from lentogenic strains, derived from wild birds [10]. Based on genetic and antigenic analyses of isolates obtained during several decades, the existence of at least eight different genotypes (I-VIII) has been shown [11-15]. Spatio-temporal and host-species associations are often seen inside these groups. Phylogenetic analysis based on the F-gene separates APMV-1 strains into class 1 and 2 clades, and the later into two sublineages which comprise the previously defined genotypes [16,17]. Lentogenic viruses of class 2, genotype 1, are naturally hosted by wild waterfowl and have an ecology resembling that of influenza A [18,19]. Class 1 viruses have also been recovered worldwide, mainly from wild waterfowl, and are with few exceptions of low-pathogenicity [12,19].

ND is regarded as one of the most important pathogens in the poultry industry where it has a great economic impact. Four ND outbreaks have occurred in Finland [20-22], the latest in 2004 when ND affected a flock of 12 000 turkeys (Ek-Kommonen, unpublished results), which were consequently destroyed. The need for vaccination of poultry in Finland was evaluated and Newcastle disease is currently controlled without vaccines.

The role of waterfowl in some of the endemic zoonotic virus infections has not been settled. In order to expand the knowledge of their prevalences in the Finnish waterfowl population, flavi-and alphaviruses were included in the study.

*Sindbis virus *(SINV) is a mosquito-borne virus of the genus *Alphavirus *in the family *Togaviridae*. It is known to cause epidemics in humans in Northern Europe characterized by fever, rash and polyarthritis [23]. The outbreaks appear to occur at 7-year intervals; the latest being in 2002 with 600 serologically verified human cases in Finland [24]. A high seroprevalence in resident birds can be seen one year after an outbreak [25].

The family *Flaviviridae *consists of about 70 viruses, most of which are arthropod-borne zoonotic agents. They infect a wide variety of vertebrates including mammals, avians and amphibians. *Tick-borne encephalitis virus *(TBEV) is the most important flavivirus in Europe, where it is endemic in several countries and has a significant impact on public health. The virus is maintained in ticks and wild vertebrates and transmission to humans occurs generally via tick bites [26]. *West Nile virus *is a mosquito-borne flavivirus endemic in Europe. Until recently, it was considered an Old World virus infecting predominantly humans and equines. Outbreaks of WN fever have been reported e.g. in humans in Romania 1996 [27] and in horses in France 2000 [28]. Since the outbreak in New York started in 1999, the virus has dispersed throughout North and Central America and is now endemic in most US states and Canadian provinces [29]. Disease in WNV-infected birds varies from symptomless to death, corvids (family *Corvidae*) being the most sensitive to lethal infections [30]. Wild bird infections by WNV, Usutu virus and SINV have been documented and birds are believed to be able to transmit these viruses geographically over long distances [31]. Migratory birds have also been shown to carry e.g. TBEV-infected ticks [32].

In order to address this need of wild bird surveillance, we chose to use an approach where hunters were recruited for blood and swab sample collection. In total 310 blood samples and 115 tracheal and cloacal swab samples were collected and studied in year 2006. Our main interest was to study the distribution of FLUAV and APMV-1 infections in our wild waterfowl populations. As SINV and TBEV are established zoonotic agents in Finland, the understanding of their ecology and possible links to wild waterfowl was also of special interest.

In this study, the circulation of both influenza A virus and APMV-1 in Finnish wild waterfowl was documented and isolated FLUAV and APMV-1 strains were genetically and phylogeneticaly characterized.

## Results

### Antibody and virus detection

Antibodies to influenza A were detected by a commercial competitive ELISA (FLUAcA). Out of 310 blood specimens, three samples, all from mallards (*Anas platyrhynchos*), were positive (competitive percentages <45). Two samples, one from a mallard and one from a common teal (*Anas crecca*) were regarded as borderline (competitive percentages 45–50). Examination of the 115 combined tracheal and cloacal swab samples showed that 13 samples were positive when studied by the influenza A M-gene specific real time RT-PCR (cycle threshold -values (Ct) 21.15–38.86); none of the samples were positive by H5-or H7-specific real-time RT-PCR. After inoculation of RT-PCR-positive specimen into embryonated eggs, 7 influenza virus isolates were successfully obtained. In only one of the samples (A/mallard/Finland/12072/06) could both antibodies (competitive percentage 48.8) and viral RNA (Ct-value 32.2) be detected (Table 1).

**Table 1:** Influenza A and APMV-1 positive samples.

			**INFLUENZA A**			**APMV-1**		
**Sample number**	**Scientific name**	**Species**	**RT-PCR (Ct)**	**Isolation**	**Serology**	**RT-PCR**	**Isolation**	**Serology**

199	*Anas platyrhynchos*	Mallard	nd	nd	+	nd	nd	-
301	*Anas platyrhynchos*	Mallard	nd	nd	+	nd	nd	-
12054	*Anas crecca*	Common teal	-	-	-	+	-	-
12072	*Anas platyrhynchos*	Mallard	+(32.6)	H3N8	+	-	-	-
12074	*Anas crecca*	Common teal	+(34.3)	H3N8	-	+	-	-
12075	*Anas platyrhynchos*	Mallard	-	-	+	-	-	-
12104	*Anas crecca*	Common teal	-	-	-	+	APMV-1	-
12110	*Anas platyrhynchos*	Mallard	+(37.5)	H3N8	-	-	-	-
12115	*Anas acuta*	Northern pintail	+(38.4)	-	-	-	-	-
12117	*Anser fabalis*	Bean goose	+(38.1)	-	-	-	-	-
12119	*Anas crecca*	Common teal	+(38.0)	-	-	+	APMV-1	-
12132	*Anas platyrhynchos*	Mallard	+(33.6)	H3N8	-	-	-	-
12133	*Anas platyrhynchos*	Mallard	+(38.8)	H3N8	-	-	-	-
12136	*Anas crecca*	Common teal	-	-	-	+	APMV-1	-
13153	*Anas crecca*	Common teal	-	-	+	-	-	+
13164	*Anas platyrhynchos*	Mallard	+(38.1)	-	-	-	-	+
13171	*Anas platyrhynchos*	Mallard	+(23.8)	H3N8	-	-	-	-
13176	*Anas platyrhynchos*	Mallard	+(38.7)	-	-	-	-	-
13183	*Anas platyrhynchos*	Mallard	+(21.1)	H3N8	-	-	-	-
13185	*Anas platyrhynchos*	Mallard	+(38.1)	-	-	-	-	-
13193	*Aythya ferina*	Common pochard	-	-	-	+	APMV-1	-

Positives/total			13/115	7/115	5/310	6/115	4/115	2/310

Percentage positives			11.3%	6.1%	1.6%	5.2%	3.4%	0.6%

Summary of influenza A virus and avian paramyxovirus-1 findings in the waterfowl samples. Positive samples are presented according to the detection method. nd = not done, sample not available.

In the screening for APMV infections, two samples, one from a common teal and one from a mallard, had titers of 1:40 in the hemagglutination inhibition (HI) test with APMV/Ulster antigen. Of the swab specimens, 6 were RT-PCR positive and from 4 of them, APMV-1 was successfully isolated in egg culture. Three of the isolates derived from common teals and one from a common pochard (*Aythya ferina*). None of the birds were positive in both RT-PCR and HI (Table 1).

When tested for antibodies to SINV by HI, none of the blood samples were found positive. Samples were not studied for SINV infections by PCR. However, three samples, all from mallards, reacted positively with WNV antigens in the HI test. Two of them had low titers of >1:20 while one reached a titer of 1:6120. Consequently, the sera were tested in parallel with TBEV antigen: the TBEV antibody titer was lower for each sample, with titers <1:20, <1:20 and 1:1280, respectively. None of the 100 studied swab samples were positive for flavivirus RNA by the hemi-nested RT-PCR using conserved primers covering most mosquito-borne flaviviruses and TBEV [33]. Positive WNV-RNA controls produced bands of the expected size.

### Subtyping and genetic characterization

**Figure 1:**
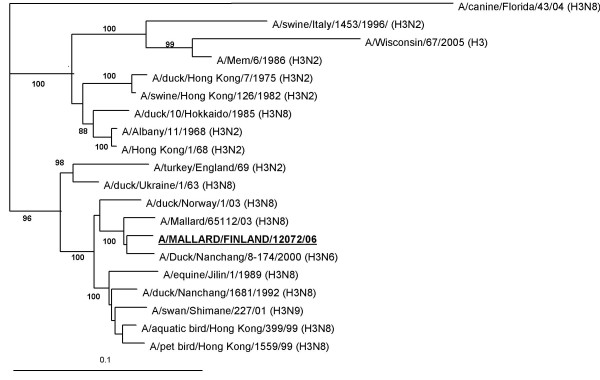
**Phylogenetic analysis of the H3 gene of A/mallard/Finland/12072/06**. Phylogenetic analysis of the H3 gene (684 nt). The tree was generated by neighbor-joining algorithm using A/canine/Florida/43/04 (H3) as outgroup. Alignments were bootstrapped 100 times. The numbers indicate confidence of analysis (bootstrap support >70% shown). Details and GenBank accession numbers to the strains are indicated in Table 2.

**Figure 2:**
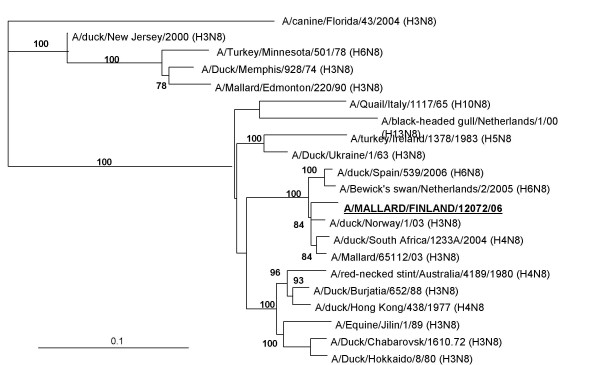
**Phylogenetic analysis of the N8 gene of A/mallard/Finland/12072/06**. Phylogenetic analysis of the N8 gene (368 nt). The tree was generated by neighbor-joining algorithm using A/canine/Florida/43/04 (N8) as outgroup. Alignments are bootstrapped 100 times. The numbers indicate confidence of analysis (bootstrap support >70% shown). Details and GenBank accession numbers to the strains are indicated in Table 3.

By serological analysis, in HI test with subtype-specific antisera, the influenza strains proved to be of the H3 subtype. Genetic analysis of the HA and NA gene sequences verified them to be of the H3N8 subtype. Nucleotide sequence alignments with the inner segment of the HA (nt 482–1166) and NA (nt 605–973) genes of the seven isolates showed that sequence identities between the isolates and the characterized strain A/mallard/Finland/12072/06 ranged from 97.2% to 99.7%. Sequence comparison revealed a close similarity (by BLAST) of the H3 gene to strains isolated from ducks in Nanchang, China [GenBank: CY006015] (97% identity) and Denmark [GenBank: AY531031] (97% identity) (Figure 1, Table 2). The closest similarity of the N8 gene was likewise to the Danish strain [GenBank: AY531032] (97% identity) and a Norwegian strain [GenBank: AJ841294] (97% identity) (Figure 2, Table 3). Both genes of A/mallard/Finland/12072/06 clustered phylogeneticaly together with mainly Eurasian strains.

**Table 2:** GenBank accession numbers for strains used in phylogenetic analysis of influenza A H3 gene.

**GenBank**	**Designation**	**Country of origin**	**Host**
AB289341	A/swan/Shimane/227/01 (H3N9)	Japan	Swan
AF348177	A/Hong Kong/1/68 (H3N2)	Hong Kong	Human
AJ427297	A/aquatic bird/Hong Kong/399/99 (H3N8)	Hong Kong	Aquatic bird
AJ427304	A/pet bird/Hong Kong/1559/99 (H3N8)	Hong Kong	Pet bird
AJ841293	A/duck/Norway/1/03 (H3N8)	Norway	Duck
AY531031	A/Mallard/65112/03 (H3N8)	Denmark	Mallard
AY531037	A/turkey/England/69 (H3N2)	Great Britain	Turkey
CY006016	A/duck/Nanchang/1681/1992 (H3N8)	China	Duck
CY006015	A/Duck/Nanchang/8-174/2000 (H3N6)	China	Duck
CY006026	A/duck/Hong Kong/7/1975 (H3N2)	Hong Kong	Duck
CY019891	A/Albany/11/1968 (H3N2)	Albany	Human
DQ124190	A/canine/Florida/43/04 (H3N8)	USA	Canine
DQ975261	A/swine/Italy/1453/1996 (H3N2)	Italy	Swine
EF473424.	A/Wisconsin/67/2005 (H3)	USA	Human
M16743	A/duck/10/Hokkaido/1985 (H3N8)	Japan	Duck
M19056.	A/swine/Hong Kong/126/1982 (H3N2)	Hong Kong	Swine
M21648	A/Mem/6/1986 (H3N2)	USA	Human
M65018	A/equine/Jilin/1/1989 (H3N8)	China	Equine
V01087	A/duck/Ukraine/1/63 (H3)	Ukraine	Duck
EU493448*	A/mallard/Finland/12072/06/H3	Finland	Mallard

* GenBank accession number for sequences from isolates obtained in this study

**Table 3:** GenBank accession numbers for strains used in phylogenetic analysis of influenza A N8 gene.

**GenBank**	**Designation**	**Country of origin**	**Host**
AB289332	A/duck/Hong Kong/438/1977 (H4N8)	Hong Kong	Duck
AJ841294	A/duck/Norway/1/03 (H3N8)	Norway	Duck
AM706354	A/duck/Spain/539/2006 (H6N8)	Spain	Duck
AY531032	A/Mallard/65112/03 (H3N8)	Denmark	Mallard
AY684900	A/black-headed gull/Netherlands/1/00	The Netherlands	Gull
AY738457	A/duck/New Jersey/2000 (H3N8)	USA	Duck
CY014631	A/red-necked stint/Australia/4189/1980 (H4N8)	Australia	Red-necked stint
CY015091	A/turkey/Ireland/1378/1983 (H5N8)	Ireland	Turkey
DQ124151	A/canine/Florida/43/2004 (H3N8)	USA	Canine
DQ822200	A/Bewick's swan/Netherlands/2/2005 (H6N8)	The Netherlands	Swan
EF041497	A/duck/South Africa/1233A/2004 (H4N8)	South Africa	Duck
L06572	A/Duck/Burjatia/652/88 (H3N8)	Russian Federation	Duck
L06573	A/Duck/Chabarovsk/1610.72 (H3N8)	Russian Federation	Duck
L06574	A/Duck/Hokkaido/8/80 (H3N8)	Japan	Duck
L06575	A/Duck/Memphis/928/74 (H3N8)	USA	Duck
L06576	A/Duck/Ukraine/1/63 (H3N8)	Ukraine	Duck
L06579	A/Equine/Jilin/1/89 (H3N8)	China	Equine
L06586	A/Mallard/Edmonton/220/90 (H3N8)	USA	Mallard
L06587	A/Quail/Italy/1117/65 (H10N8)	Italy	Quail
L06588	A/Turkey/Minnesota/501/78 (H6N8)	USA	Turkey
EU493449*	A/mallard/Finland/12072/06/N8	Finland	Mallard

* GenBank accession number for sequences from isolates obtained in this study.

**Figure 3:**
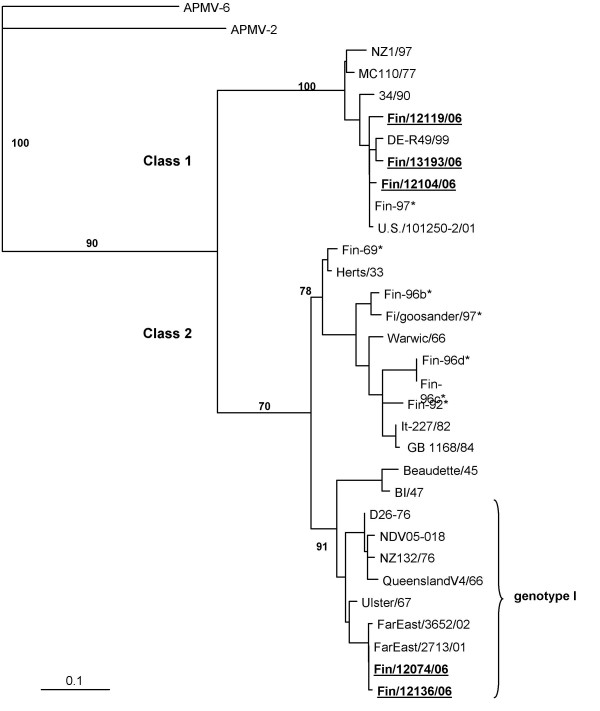
**Phylogenetic analysis of APMV-1 isolates**. Phylogenetic analysis of the F-gene cleavage site (208 nt) of strains isolated in Finland in 2006. The tree was generated by neighbor-joining algorithm using APMV-2 and APMV-6 as outgroups. Alignments are bootstrapped 500 times. The numbers indicate confidence of analysis. Previous Finnish isolates are marked with *. Details and GenBank accession numbers to the strains are indicated in Table 4.

Sequences of the F genes of the APMV-1 isolates revealed that the isolates were of two different lineages (Figure 3, Table 4): three isolates had a high similarity (98–99% identity by BLAST) to strain FIN-97 [GenBank: AY034801], a previous Finnish isolate, and to the North American strain US/101250-2/01 [GenBank: AY626268], of class 1. One isolate and one sample only positive by RT-PCR were most similar to Far Eastern isolates [GenBank: AY965079, AY972101] (99% identity) and had 96% similarity to strain Ulster/67 [GenBank: AY562991] representing class 2, genotype I.

**Table 4:** GenBank accession numbers for strains used in phylogenetic analysis of APMV-1 isolates.

**GenBank**	**Designation**	**Country of origin**	**Host**
AF003726	MC110/77	France	Shelduck
AF003727	34/90	Ireland	Chicken
AF091623	Fi/goosander/1997	Finland	Goosander
AF109885	GB 1168/84	Great Britain	Pigeon
AF438366	NZ132/76	New Zealand	Mallard
AF438370	NZ1/97	New Zealand	Mallard
AJ880277	It-227/82	Italy	Pigeon
AY029299	APMV-6	Taiwan	Duck
AY034794	Fin-69	Finland	Willow grouse
AY034796	Fin-92	Finland	Pigeon
AY034798	Fin-96b	Finland	Goosander
AY034799	Fin-96c	Finland	Pigeon
AY034800	Fin-96d	Finland	Pigeon
AY034801	Fin-97	Finland	Mallard
AY741404	Herts/33	Great Britain	Chicken
AY562991	Ulster/67	Ireland	Chicken
AY626268	U.S./101250-2/2001	USA	Chicken
AY965079	FarEast/2713/2001	Russian Federation	Duck
AY972101	FarEast/3652/2002	Russian Federation	Duck
D13977	APMV-2, Yucopa	USA	Chicken
DQ097393	DE-R49/99	Germany	Duck
DQ439875	NDV05-018	China	Chicken
M24692	D26-76	Japan	Chicken
M24693	QueenslandV4/66	Australia	Chicken
M24695	BI/47	USA	Chicken
X04719	Beaudette/45	USA	Chicken
Z12111	Warwic/66	Great Britain	Chicken
EU493450*	APMV-1/teal/Finland/12074/06	Finland	Teal
EU493451*	APMV-1/teal/Finland/12104/06	Finland	Teal
EU493452*	APMV-1/teal/Finland/12119/06	Finland	Teal
EU493453*	APMV-1/teal/Finland/12136/06	Finland	Teal
EU493454*	APMV-1/pochard/Finland/13193/06	Finland	Common pochard

* GenBank accession numbers for sequences from isolates obtained in this study

The cleavage site of the fusion (F) protein has been generally used as an indicator for pathogenicity. Velogenic strains possess at least two basic amino acids immediately surrounding glutamine 114 while lentogenic strains lack this domain [34,35]. Our strains had either the cleavage site sequence SGGERQERLVG or SGGGKQGRLIG, both typically found in lentogenic strains (Table 5).

**Table 5:** Characterization of avian paramyxovirus-1 isolates.

**Isolate**	**Host**	**F protein cleavage site**	**Class [16,17]**	**Genotype [15]**
Fin/12074/06	*Anas crecca*	SGGGKQGRLIG	2	I
Fin/12104/06	*Anas crecca*	SGGERQERLVG	1	VI
Fin/12119/06	*Anas crecca*	SGGERQERLVG	1	VI
Fin/12136/06	*Anas crecca*	SGGGKQGRLIG	2	I
Fin/13193/06	*Aythya ferina*	SGGERQERLVG	1	VI

Legend to Table 2: Characterization of the APMV-1 isolates. Amino acid sequences at the fusion protein cleavage site (amino acids at position 109–119) and classification of the strains are indicated.

The sequences obtained from the isolates described in this study have been submitted to GenBank with the accession numbers listed in Tables 2, 3, 4.

## Discussion

The circulation of influenza A viruses in the Finnish waterfowl population in fall 2006 was shown in this study; no viruses of the potentially highly pathogenic H5 or H7 subtypes could be detected. According to the M-gene real-time RT-PCR, the prevalence of influenza A viruses was 11.3% (n = 115) in all analysed birds, 16.3% (n = 55) in all analysed mallards (*Anas platyrhynchos*) and 5.4% (n = 37) in all analysed teals (*Anas crecca*). These values correspond well with previous studies where extensive studies on wild waterfowl in Sweden have shown a 14.5% prevalence of FLUAV during fall, when the prevalence appears to be highest [36]. Although influenza A viruses replicate mainly in the intestinal tract and are shed with feces to wading waters [37], recently it has been suggested that at least some of the HPAI strains are preferentially recovered from tracheal specimen. Whether the viral RNA obtained in our study was recovered from tracheal or from cloacal specimen remains unknown as these were pooled together. It is also noteworthy that the viral load estimated by real-time RT-PCR varied considerably in the 7/13 FLUAV isolation positive samples: two samples were strongly positive (Ct 21–24) while five samples were much weaker positives (Ct >32, two of these Ct >37). The prevalence of infection of FLUAV when studied by the presence of specific antibodies by a commercial competitive ELISA was only 1.6% (n = 310). Screening of antibodies in this format does not seem efficient or sensitive for detection of prevalence of infection.

**Figure 4:**
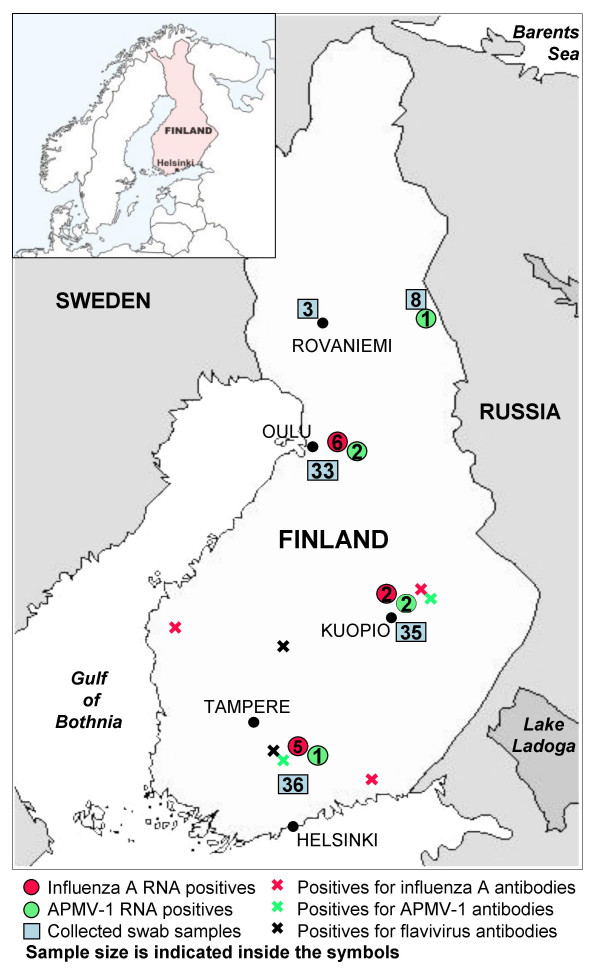
**Geographic distribution of collected samples**. The squares indicate the total sample size and circles PCR-positive samples. Antibody findings are indicated with a cross. Each virus is marked with its own color.

The subtype diversity of circulating avian influenza viruses in Europe and Asia during the past few years has been extensive, as summarized by Alexander [38], however, only one subtype (H3N8) was recovered in this study. In a Swedish study based on material collected during the years 2002–2004, 11 different HA subtypes and all 9 NA subtypes were found [36]. Out of 129 isolates only 5 were of the H3N8 subtype while in the North American study, described by Krauss et al., viruses of the H3N8 subtype were most commonly found (22.8% of isolates from ducks) in the 16-year study [39]. Other recent H3N8 findings have been reported from Denmark in 2003 [40] and Norway in 2005 [41]. As we have not found any methodological reasons to explain the subtype homogeneity of our findings, the results could be explained by the limited time period of sample collection; birds were sampled during one hunting season of only a few months and from a limited number of sampling sites; the material represented only few duck populations (Figure 4). It could also be simply due to the seasonality of subtype prevalences. All H3N8 isolates, except one from a teal, were derived from mallards.

To conclude, of our 115 swab samples 13 were influenza A RT-PCR positive and of those samples 7 viruses were isolated. In 2006 HPAI H5N1 viruses occurred widely in birds in Europe [38] but were not reported from Finland. Our results, with H3N8 as the only detected subtype, support the view that this subtype was indeed absent at that time.

There have been occasional isolations of APMV-1 in Finland from birds representing different orders, e.g. pigeons (*Columbidae*), pheasants (*Phasianidae*) and goosander (*Mergus merganser*). Antigenic and genetic analysis of viruses isolated from three outbreaks in pheasants in Denmark between August and November 1996, from a goosander in Finland in September 1996, from an outbreak in chickens (*Gallus gallus*) in Norway in February 1997 and from an outbreak in chickens in Sweden 1997 indicate that they were all essentially similar. The results are consistent with the theory that the virus was introduced to the different locations by migratory birds [42]. The latest outbreak in poultry occurred in July 2004 when APMV-1 was isolated from turkeys (*Meleagrididae*) on a farm in Finland. The pathogenicity index was verified by VLA (Weybridge, UK) to be >0.7 and the virus was thereby classified as Newcastle disease virus. The birds were destroyed and the outbreak was handled accordingly. Interestingly, ND was reported from two sites in Sweden at the same time, but no connection to the Finnish outbreak was found. According to VLA reports (Veterinary Laboratories Agency, Weybridge, UK), virus isolates from all three sites were highly similar. The origin of the Finnish outbreak was never found but wild birds were suspected.

The prevalence of APMV-1 was 5.2% (n = 115) in our study. Five of the six RT-PCR positive samples came from common teal, although teals represented only 32.2% of our material. One isolate derived from the only pochard (*Aythya ferina*) sampled in this study. Two teals appeared to be infected with both FLUAV and APMV-1.

Based on genetic characterization, our isolates clustered into two distinct lineages (Figure 3). Three isolates (Fin/12104/06, Fin/12119/06 and Fin/13193/06) were of class 1, which represents mainly avirulent viruses found worldwide from wild waterfowl, including the lentogenic strain MC110/77 and velogenic strain 34/90 [12]. The global distribution of the class 1 strains is also seen in the clustering of our isolates with geographically distant isolates. Our isolates were obtained from different sites in North, Central and South Finland, suggesting that viruses of this lineage are dispersed through the country (Figure 4). Interestingly, isolates obtained in a recent North American study [19] of APMV-1 in waterfowl and shorebirds showed high sequence similarity (97–98%) to our class 1 isolates (data not shown).

Two isolates (Fin/12074/06 and Fin/12136/06) were of class 2, genotype I, which includes Ulster-like viruses. Finnish APMV-1 isolates have been previously characterized [22], and this is the first time that viruses of genotype I have been found (Figure 3). These two isolates were also derived from different regions. Generally viruses of genotype I cause little or no disease in poultry, and derivatives, e.g. Ulster2C/67 and Queensland/V4, have been used as live vaccines in many countries. Avirulent strains have been isolated worldwide in waterfowl but have occasionally been linked to virulent disease outbreaks, e.g. 1998–2000 in Australia [43].

Two basic amino acid pairs surrounding the fusion protein cleavage site usually indicate increased virulence [44]. Analysis of the amino acid sequence of the F-protein cleavage site (109–119) showed all of the isolates to be of avirulent type lacking the basic amino acids (Table 5). Other paramyxovirus types (APMV-2-9) were not studied but these findings show that type 1 avian paramyxovirus is probably endemic in the Finnish waterfowl populations.

None of the samples were positive for Sindbis virus antibodies in the HI test. Previous studies in Finland have demonstrated SINV antibodies in resident grouse (*Tetraonidae*) with a possibly cyclic pattern. The total prevalence of SINV HI antibodies was 27.4 % in 2003 and dropped down to 1.4 % in 2004 [25]. Wild tetraonid and passerine birds have been suggested to play a role as amplifying hosts and some migratory birds are known to be able to distribute SINV over long distances [45,46]. In this study, evidence of the involvement of wild waterfowl in the ecology of SINV was not found.

We found three mallard samples reactive against WNV antigen in HI test, one of which had a significantly high titer of 1/6120. The lower HI titers towards TBEV are suggestive for antibody specificity against a mosquito-borne flavivirus, however these results require further confirmation by neutralization test [47]. Although previous studies have shown serological evidence of West Nile virus infections in birds in Germany [48], Hungary [49], Poland [50] and the UK [31], to our knowledge, mosquito-borne flavivirus infections have not been reported from Northern Europe. It is possible that migratory birds arriving annually from endemic areas to Finland could carry and transmit mosquito-borne flaviviruses through ornithophilic mosquitoes.

Finally, the involvement of hunters in the sampling of wild waterfowl was found to be a suitable way to screen birds. The percentage of different species in our material (Table 6) correlates well with the percentage of the same species in the nationwide waterfowl bag in 2006 (total bag 552 600 individuals) [51]. For example, the four most numerous species in our sample jointly represented 92% of the birds in the nationwide bag, mallard (51%) and teal (21%) being the most numerous bagged species. Most of the sampled birds had presumably migrated from the east (Russia) as only about 200 000 pairs of both mallard and teal are estimated to nest in Finland.

**Table 6:** Samples and species.

**Species**		**Blood samples (%)**	**Swab samples (%)**
Mallard	*Anas platyrhynchos*	155 (50.0)	55 (47.8)
Common teal	*Anas crecca*	84 (27.1)	37 (32.2)
Eurasian wigeon	*Anas penelope*	23 (7.4)	6 (5.2)
Common goldeneye	*Bucephala clangula*	16 (5.2)	7 (6.1)
Greylag goose	*Anser anser*	11 (3.5)	0
Northern pintail	*Anas acuta*	6 (1.9)	6 (5.2)
Bean goose	*Anser fabalis*	5 (1.6)	2 (1.7)
Garganey	*Anas querquedula*	4 (1.3)	0
Tufted duck	*Aythya fuligula*	3 (1.0)	1 (0.9)
Red-breasted merganser	*Mergus serrator*	2 (0.6)	0
Common pochard	*Aythya ferina*	1 (0.3)	1 (0.9)

Total		310	115

Legend to Table 6: The sample size of blood samples collected on filterpaper strips and combined tracheal and cloacal swabs by bird species.

## Conclusion

Circulation of both influenza A virus and APMV-1 in Finnish wild waterfowl was documented in this study with prevalences of 11.3% and 5.2%, respectively. The subtype H3N8 was the only subtype of influenza A detected while the APMV-1 viruses detected represented two distinct genetic groups, class 1 and class 2, genotype 1. The results suggest that both the sampling and detection methods were effective, and the methods would likely have detected e.g. HPAI H5N1 infections occuring in poultry and wild birds in other European countries in 2006. Screening of antibodies was less efficient in detecting the prevalence of infection. Notably, serological evidence of flavivirus infection in wild waterfowl in Finland was documented.

## Methods

### Sample overview

We tested 310 blood samples and 115 mixed tracheal and cloacal swabs from birds representing 11 different species belonging to the order *Anseriformes*. Mallards (*Anas platyrhynchos*) were by number the best-represented species, with 50.0% of the blood samples and 47.8 % of the swab samples. Teals (*Anas crecca*) counted for 27.1% and 32.2% of the respective sample types (Table 6). The samples were collected by hunters during the annual duck hunting season preceding peak fall migration. All samples were collected during an 8-week time-period starting 20th August 2006. The blood sampling covered the whole country while the swab samples were collected from three main areas (Figure 4).

### Sample collection

Hunters were asked to collect blood samples (preferentially from the heart) from hunted waterfowl on filter-paper strips and to enclose the dried samples individually in airtight plastic bags. Samples were sent to the Department of Virology, University of Helsinki. The hunters were asked to identify the species and mark the location and date of collection for each sample. The samples were diluted immediately upon arrival or stored at -20°C. Approximately 1 cm^2 ^of blood-stained filter paper was sliced and blood was eluted in 1 ml of Dulbecco's phosphate buffered saline with 0.2% bovine albumin serum to a final concentration of approximately 1:10 [24,25]. Aliquots were stored at -20°C until tested.

Hunters and staff from the Finnish Game and Fisheries Research Institute (RKTL) collected swab samples using commercial nylon-flocked swabs which were placed in tubes containing 1 ml Universal Transport Medium (Copan Innovation, Brescia, Italy). Samples were kept frozen at -20°C until transferred to the laboratory at The Finnish Food Safety Authority (Evira, Helsinki) where they were stored at -80°C until tested. From each bird, tracheal and cloacal swab samples were taken and placed in the same tube. A corresponding blood sample (collected as described above) was available for each of the 115 mixed swab samples.

### Serological examination

Blood samples were tested for antibodies to influenza A with the commercial FLUAcA competitive ELISA Kit based on the nucleocapsid protein (ID.VET, Montpellier, France). Samples were inactivated for 30 minutes in a +56°C water bath prior to testing. The competition percentages were calculated according to the manufacturer's recommendations by dividing the sample OD measured at 450 nm with the negative control OD and multiplying the sum with 100. Percentages less than or equal to 45 were considered positive, percentages higher than or equal to 50 negative and percentages between 45 and 50 borderline. Samples with a competition percentage less than 55 were re-examined.

Blood samples were examined individually for antibodies to APMV-1, SINV and flaviviruses by hemagglutination inhibition test (HI). Prior to testing, for HI microtitration with SINV and flavivirus antigens, the diluted serum samples were absorbed with kaolin and male goose erythrocytes. For microtitration with APMV-1 antigen the serum samples were inactivated for 30 minutes in a +56°C water bath.

Blood samples were screened for antibodies by HI test using WNV, SINV and APMV-1/Ulster antigens. As all the viruses in the flavivirus group are cross-reactive in HI, West Nile virus-positive samples were further analysed in a parallel HI test with both WNV and TBEV antigen. The SINV, WNV and TBEV strains used were inactivated with Tween-ether and APMV-1 with formaldehyde. Human seropositive and seronegative sera were used as controls for SINV, WNV and TBEV. For APMV-1 a positive turkey serum was used as control. The HI was performed with two-fold dilutions starting from 1:20. The protocol for the detection of arbovirus antibodies by HI has been described previously [52-54]. The protocol for the detection of APMV-1 infection was adapted from Council Directive 92/66/EEC Annex III [55], with the exception that rooster erythrocytes were used instead of chicken erythrocytes. The APMV-1 controls and NDV/Ulster antigen were obtained from VLA (Weybridge, UK).

### RNA extraction and RT-PCR

RNA was extracted from swab samples using semi-automated ABI PRISM™ 6100 Nucleic Acid PrepStation and reagents (Applied Biosystems, Foster City, CA, USA). Influenza A viruses were detected by a real time RT-PCR assay targeting the highly conserved matrix gene [56]. Samples with cycle threshold (Ct) values <40 were further studied with a real time RT-PCR targeting the H5 and H7 genes [57]. In order to detect as many strains as possible, two different primer pairs were used for APMV nucleic acid amplification by RT-PCR (Table 7), described previously by Seal et al. [58] and Huovilainen et al. [22]. Both primer pairs target the F-gene and include the fusion protein cleavage site.

**Table 7:** Primers used for influenza A and avian paramyxovirus-1 amplification.

**Gene**	**Forward primer**	**Reverse primer**
AIV HA	**ha1 [59]**	**h3a**
	TAT TCG TCT CAG GGA GCA AAA GCA GGG G	TTG TCA AAA TTG TCA TTG TTT GG
	**h3c**	**h3b**
	GCA AAA GGG GAC CTG CTA G	TTC CCA TTG ATC TGG TCA ATG
	**h3d**	**ha2 [59]**
	TCA GGC ATC AAA ATT CCG AAG	ATA TCG TCT CGT ATT AGT AGA AAC AAG GGT GTT TT
AIV NA	**na1 [59]**	**n8a**
	TAT TGG TCT CAG GGA GCA AAA GCA GGA GT	GGA ATT AAT GAC GTC AGT AGG
	**n8b**	**n8c**
	GCC TGA TTC CAA AGC AGT AG	GTT GGG TAT TTA TGT GCA GGG
	**n8c**	**NA2 [59]**
	GTT GGG TAT TTA TGT GCA GGG	ATA TGG TCT CGT ATT AGT AGA AAC AAG GAG TTT TTT
APMV-1 F	**Fa [58]**	**Fb [58]**
	CTG CCA CTG CTA GTT GIG ATA ATC C	CCT TGG TGA ITC TAT CCG IAG
	**Fc [22]**	**Fd [22]**
	CCC TCC TTG CCC CGC TC	CTG CTG CAT CTT ACC TAC

(I stands for inosine)

RNA from 100 swab specimens were also studied by a heminested RT-PCR. The primers are according to and protocol adapted from the method described by Scaramozzino et al. [33] with minor modifications.

### Virus isolation and characterization

All samples positive in the FLUAV matrix gene real-time RT-PCR and in APMV-1 RT-PCR assays were subjected to virus isolation attempts by inoculating swab specimen into the allantoic cavity of four 8–10 day-old embryonated chicken eggs. The allantoic-amniotic fluids were harvested from eggs with dead and dying embryos as they arose and from all remaining eggs six days post-inoculation, and were tested for hemagglutinating activity. FLUAV isolates were tentatively characterized by HI test using subtype-specific polyclonal antisera obtained from VLA.

The preliminary genetic subtyping was done by sequencing the both ends of RT-PCR products of HA and NA genes as described by Hoffman et al. [59] and performing BLAST searches with the obtained data. All isolates were serologically and genetically typed as H3N8 viruses and the HA and NA genes of A/mallard/Finland/12072/06 were amplified and sequenced with three overlapping primer pairs (Table 7).

APMV-1 strains were amplified and sequenced using the PCR primers targeting the F-gene [22,58]. The 208-nucleotide-long sequences covering the fusion protein cleavage site were subjected to phylogenetic analysis and converted to amino acid sequence. The basic amino acids surrounding the fusion protein cleavage site (109–119) were studied as pathogenicity markers (Table 5).

PCR products for sequencing were extracted using Mini Gels, DNA Recovery Kit and BandPick™ (Elchrom Scientific, Cham, Switzerland). The sequencing reactions were run on Applied Biosystems 3100 Avant capillary DNA sequencer and using BigDye Terminator v3.1 chemistry (Applied Biosystems). Reaction products were purified using DyeEx 2.0 Spin Kit (Qiagen, Helsinki, Finland).

### Sequence analysis

Phylogenetic analysis was performed on the nucleotide sequences of full-length H3 and N8 genes. The middle parts of the genes were analysed for all FLUAV isolates but sequence from only one strain (A/mallard/Finland/12072/06) was used for phylogenetic analysis as the nucleotide sequence of all strains were highly similar, though not identical. Additionally, phylogenetic analysis on the partial F-gene (nt 4846–5053) of all APMV-1 isolates and the sequence obtained from one APMV-1 RT-PCR positive sample, which failed to grow, was included.

The sequences were compared with published sequences by search in EBI WU-Blast2 database [60]. For alignments and phylogenetic trees the most closely related, according to the BLAST-search, and more distant strains were chosen. Nucleotide sequences were managed within the BioEdit [61], and aligned with ClustalX [62]. Phylogenetic trees were generated by the neighbor-joining algorithm within the PHYLIP 3.67 package [63] from 100 or 500 bootstrap replicates. Maximum likelihood (PHYLIP) was used to calculate the branch lengths of the consensus trees, and these were presented graphically by the TreeView program [64].

## Competing interests

The author(s) declare that they have no competing interests.

## Authors' contributions

The study was conceived and the manuscript drafted by EL, OV and AV. AH and CE-K additionally contributed to the study design and revision of the manuscript. EL was the main author and performed serological assays, analysis and interpretation of data and sequences, and coordinated sample collection. AH provided expertise in molecular genetics and influenza A and CE-K in serology, virus isolation and in APMV-1. OR and HP coordinated sample collection and provided expertise in avian ecology. TS contributed with expertise in phylogeny and generation of phylogenetic trees and EH with development of WNV-HI. Along with study design, OV and AV provided expertise in virology and zoonotic diseases. All authors' have read and approved the final manuscript.
